# A Case of Advanced Descending Colon Cancer in an Adult Patient with Intestinal Malrotation

**DOI:** 10.1155/2016/3194056

**Published:** 2016-03-03

**Authors:** Yoshifumi Nakayama, Masaki Akiyama, Yusuke Sawatsubashi, Noritaka Minagawa, Takayuki Torigoe, Keiji Hirata

**Affiliations:** ^1^Department of Surgery 1, School of Medicine, University of Occupational and Environmental Health, 1-1 Iseigaoka, Yahata-nishi-ku, Kitakyushu 807-8555, Japan; ^2^Department of Gastroenterological and General Surgery, Wakamatsu Hospital of University of Occupational and Environmental Health, 1-17-1 Hamamachi, Wakamatsu-ku, Kitakyushu 808-0024, Japan

## Abstract

This report presents an operative case of advanced descending colon cancer in an adult patient with intestinal malrotation. A 63-year-old Japanese male was suffering from left side abdominal pain, abdominal distension, and constipation. An endoscopic examination revealed an advanced tumor in the descending colon. Computed tomography (CT) of the abdomen revealed the thickening of the descending colon wall and superior mesenteric vein rotation. An opaque enema detected severe stenosis of the descending colon. An abdominal X-ray examination revealed the dilation of the colon and small intestine with niveau. At the insertion of an ileus tube, the C-loop of the duodenum was observed to be absent and the small intestine was located on the right side of the abdomen. After the decompression of the bowel contents, laparotomy was performed. Descending colon cancer was observed to have directly invaded the left side of the transverse colon. Left hemicolectomy, lymph node dissection, and appendectomy were performed. The patient had an uneventful recovery and was discharged from the hospital on the 16th day after surgery. This report presents a rare operative case of descending colon cancer in an adult patient with intestinal malrotation.

## 1. Introduction

Intestinal malrotation can be defined as any deviation from the normal 270-degree rotation of the midgut in a counterclockwise direction during embryonic development [1]. Malrotation or nonrotation of the midgut occurs in approximately 1 in 500 live births [1]. Intestinal malrotation is diagnosed during the first month of life in 85–90% of all cases [2]. Intestinal malrotation is rarely present in adulthood. A barium enema study of 2000 adults demonstrated that the prevalence of malrotation was 0.2% [3]. Synchronous colon cancer in adult patients with intestinal malrotation is extremely rare [4].

This report presents a rare case of descending colon cancer in an adult patient with intestinal malrotation.

## 2. Case Report

A 63-year-old Japanese male presented with the chief complaints of left side abdominal pain, abdominal distension, and constipation. As the patient's symptoms worsened, he underwent an endoscopic examination, which revealed the presence of an ulcerated tumor of the descending colon. He was referred to the surgical outpatient clinic of our hospital with advanced descending colon cancer.

**Figure 1:**
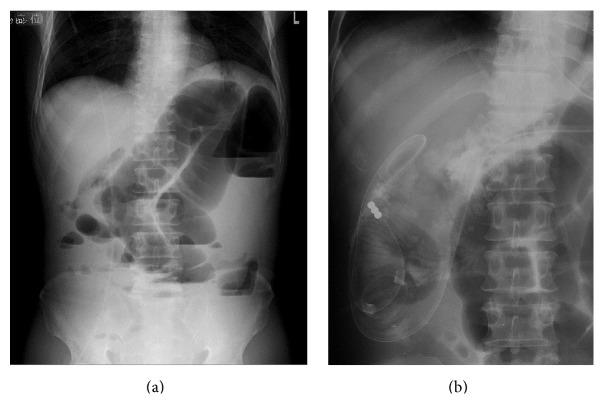
An abdominal X-ray examination revealed the dilation of the colon and niveau (a). At the insertion of an ileus tube, the C-loop of the duodenum was observed to be absent and the small intestine was located on the right side of the abdomen (b).

**Figure 2:**
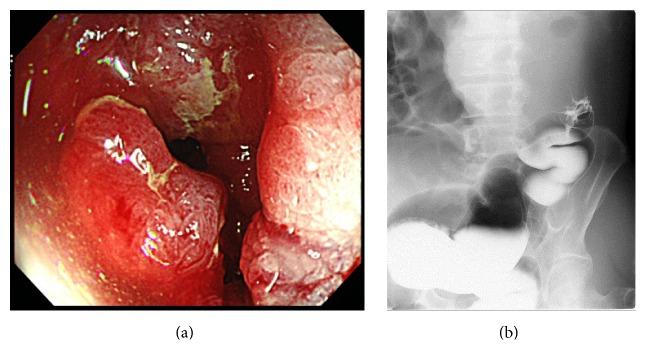
Colonoscopy revealed an advanced tumor in the descending colon (a). An opaque enema using contrast fluid revealed severe stenosis of the descending colon. The contrast fluid could not pass the stenotic part of the descending colon (b).

**Figure 3:**
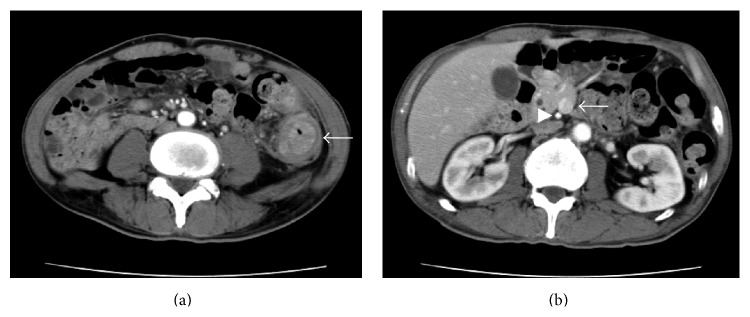
Computed tomography (CT) of the abdomen revealed thickening of the descending colon wall, which was indicative of advanced descending colon cancer (arrow) (a); superior mesenteric vein rotation was also observed (arrow) (b).

**Figure 4:**
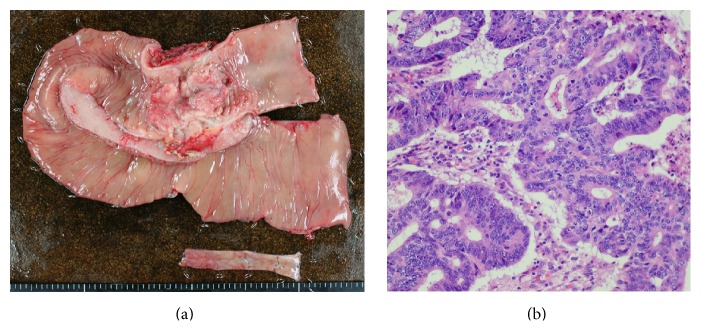
An operative specimen revealed advanced descending colon cancer with direct invasion of the transverse colon (a). A histopathological examination with hematoxylin and eosin staining confirmed that the lesion was moderately differentiated tubular adenocarcinoma (b).

The laboratory data showed the patient had a white blood cell count of 6,200/mm^3^, a hemoglobin level of 8.7 g/dL, a hematocrit level of 25.3%, and a platelet count of 401,000/mm^3^. The patient's electrolyte and blood urea nitrogen levels were normal, as was his liver function. His CEA and CA19-9 levels were 1.7 ng/mL and CA19-9 was 21.3 U/mL, respectively. An abdominal X-ray examination revealed a dilated colon and niveau (Figure 1(a)). These findings were consistent with bowel obstruction. Computed tomography (CT) of the abdomen revealed the thickening of the descending colon wall, which was indicative of advanced descending colon cancer (Figure 3(a)); superior mesenteric vein rotation was also observed (Figure 3(b)). Colonoscopy revealed an advanced tumor in the descending colon (Figure 2(a)). The biopsy specimen was found to be moderately differentiated tubular adenocarcinoma. An opaque enema, using contrast fluid, revealed severe stenosis of the descending colon. The contrast fluid could not pass the stenosis (Figure 2(b)). At the insertion of an ileus tube, the C-loop of the duodenum was observed to be absent and the small intestine was located on the right side of the abdomen (Figure 1(b)). After the decompression of the bowel contents, laparotomy was performed. The small intestine was located on the right side of the abdomen. The appendix, cecum, and ascending colon were located in the middle abdomen. Descending colon cancer was observed to have directly invaded the left side of the transverse colon (Figure 4(a)). Left hemicolectomy, lymph node dissection, and appendectomy were performed. A histopathological examination with hematoxylin and eosin staining of the resected lesion revealed moderately differentiated adenocarcinoma (Figure 4(b)). No metastatic regional lymph nodes were identified. However, some lymphatic and venous invasion of the carcinoma cells was observed.

The patient had an uneventful recovery and was discharged from the hospital on the 16th day after surgery. He has been followed up in the outpatient clinic without recurrence for approximately 9 years.

## 3. Discussion

Given that intestinal malrotation in adults is not common and is usually asymptomatic [3], the clinical importance of intestinal malrotation in adults is less well documented than in children. Most of the clinical symptoms of this disorder are due to the presence of coincidental intestinal disease. Many cases of adult intestinal malrotation are only discovered coincidentally at surgery for other diseases. In the present case, clinical symptoms of the patient, which included abdominal pain, abdominal distension, and constipation, occurred due to descending colon cancer with bowel obstruction, which was coincidental with the intestinal malrotation.

Intestinal malrotation has been divided into four types: nonrotation type, malrotation type, reversed rotation type, and paraduodenal hernia type, according to the arrest point of development (during embryonic development) anywhere along the rotation of the midgut in a counterclockwise direction [17]. Kato et al. summarized the Japanese literature and reported the frequency of each of the types of adult intestinal malrotation (nonrotation type (53.3%), malrotation type (31.1%), reversed rotation type (6.7%), and paraduodenal hernia type (8.9%)) [18]. The present case seemed to correspond to the nonrotation type, which is the most frequent one. Moreover, in 82.8% of the colorectal cancer patients with intestinal malrotation, the malrotation was classified as nonrotation type [19].  Table 1 shows that nonrotation type malrotation occurred most frequently (75.0%) in colorectal cancer patients with intestinal malrotation.

Table 1 summarized the cases of colon cancer in adult patients with intestinal malrotation that are reported in the English literature, excepting the coexistent cases with situs inversus totalis [4–16]. Most of the patients had right-sided colon cancer (Table 1). Left-sided colon cancer is rare in adult patients with intestinal malrotation. The rate of left-sided colon cancer was 14.3% (Table 1). Anatomical disorders of the placement in this type of intestinal malrotation may be associated with carcinogenesis in the colon. Ren and Lu suggested that malrotation of the gut which may cause chronic bowel obstruction may lead to inflammation and carcinogenesis [8].

There are some problems at the operation in the cases of synchronous colon cancer in adult patients with intestinal malrotation. One of these problems is arterial and venous variations. Before the operation, confirm the presence of arterial and venous variations via enhanced CT. In the case of our patient, superior mesenteric vein rotation (Figure 3(b)) and anomalies were identified. It is important to understand vessel anomalies on CT angiography or enhanced CT scan before surgery [19]. Based on the imaging information, the regional lymph node dissection could be performed along with the arteries.

We herein presented a rare operative case of descending colon cancer in a patient with intestinal malrotation.

## Figures and Tables

**Table 1:** Case reports of colon cancer in adult patient with intestinal malrotation in English literature, which except the coexistent cases with situs inversus totalis.

Number	Citation including year	Age	Sex	Location	Histologic type	Type of IM
1	Gilbert et al., 1990 [5]	55	M	Splenic flexure	Adenoca.	Nonrotation
2	Torreggiani et al., 2001 [6]	86	F	Cecum	Carcinoma	Nonrotation
3	Uchida et al., 2004 [7]	57	M	Transverse colon		Nonrotation
4	Ren and Lu, 2009 [8]	45	M	Ascending colon	Well to mod. diff.	Nonrotation
5	Brillantino et al., 2009 [9]	34	M	Cecum	Well diff.	Nonrotation
6	Michalopoulos et al., 2010 [10]	76	M	Ascending colon	Neoplasm	Reversed rotation
7	Morimoto et al., 2012 [11]	57	M	Cecum	Cancer	Reversed rotation
8	Donaire et al., 2013 [12]	52	M	Right colon	Adenoca.	Nonrotation
9	Hirano et al., 2013 [13]	68	F	Ascending colon	Well diff.	Nonrotation
10	Hirano et al., 2013 [14]	82	F	Transverse colon	Well diff.	Reversed rotation
11	Enomoto et al., 2014 [15]	48	M	Transverse colon	Well to mod. diff.	Nonrotation
12	Lu, 2014 [4]	43	F	Cecum	Adenosquamous ca.	NA
13	Ray et al., 2014 [16]	60	F	Ascending colon	Mod. diff.	NA
14	Our case, 2015	63	M	Descending colon	Mod. diff.	Nonrotation

IM: intestinal malrotation. NA: no assessment.
